# Genome-wide identification of long intergenic non-coding RNAs of responsive to powdery mildew stress in wheat (*Triticum aestivum*)

**DOI:** 10.3389/fpls.2023.1297580

**Published:** 2023-11-24

**Authors:** Peina Cao, Youning Wang, Zhaolan Ma, Xiao Xu, Dongfang Ma, Lijun Yang

**Affiliations:** ^1^ Engineering Research Center of Ecology and Agricultural Use of Wetland, Ministry of College of Agriculture, Yangtze University, Jingzhou, China; ^2^ Hubei Key Laboratory of Quality Control of Characteristic Fruits and Vegetables, Hubei Engineering University, Xiaogan, Hubei, China; ^3^ Jiangsu Academy of Agricultural Sciences, Jiangsu Coastal Area Institute of Agricultural Sciences, Yancheng, China; ^4^ Key Laboratory of Integrated Pest Management on Crop in Central China, Ministry of Agriculture/Hubei Province Key Laboratory for Control of Crop Diseases, Pest and Weeds/Institute of Plant Protection and Soil Science, Hubei Academy of Agricultural Sciences, Wuhan, Hubei, China

**Keywords:** wheat, powdery mildew, lincRNAs, expression pattern, differential expression

## Abstract

Wheat powdery mildew caused by *Blumeria graminis* f. sp. *tritici* is one of the most serious foliar diseases of wheat, causing grain yield and quality degradation by affecting plant photosynthesis. It is an effective method to improve the disease resistance of wheat plants by molecular breeding. With the continuous development of sequencing technology, long intergenic noncoding RNAs (lincRNAs) have been discovered in many eukaryotes and act as key regulators of many cellular processes. In this study, 12 sets of RNA-seq data from wheat leaves pre- and post-pathogen infection were analyzed and 2,266 candidate lincRNAs were identified. Consistent with previous findings, lincRNA has shorter length and fewer exons than mRNA. The results of differential expression analysis showed that 486 DE-lincRNAs were selected as lincRNAs that could respond to powdery mildew stress. Since lincRNAs may be functionally related to their adjacent target genes, the target genes of these lincRNAs were predicted, and the GO and KEGG functional annotations of the predicted target genes were performed. Integrating the functions of target genes and the biological processes in which they were involved uncovered 23 lincRNAs that may promote or inhibit the occurrence of wheat powdery mildew. Co-expression patterns of lincRNAs with their adjacent mRNAs showed that some lincRNAs showed significant correlation with the expression patterns of their potential target genes. These suggested an involvement of lincRNAs in pathogen stress response, which will provide a further understanding of the pathogenic mechanism of wheat powdery mildew.

## Introduction

Noncoding RNAs (ncRNAs) have emerged as major components of the eukaryotic transcriptome compared to protein-coding genes (Ariel et al., 2015). The role of ncRNAs as potent and specific regulators of gene expression is now widely recognized in almost all species to date (Brosnan and Voinnet, 2009; Beermann et al., 2016; Yamada, 2017). The long non-coding RNAs (lncRNAs) are the largest family of the ncRNAs. There are three kinds of lncRNAs: long intergenic non-coding RNAs (lincRNAs), intronic lncRNAs, and antisense lncRNAs (Chen and Zhu, 2022). Based on the location and length information, long intergenic non-coding RNAs (lincRNAs, a type of lncRNA), longer than 200 nucleotides (nt), are an abundant class of endogenous RNA molecules that are transcribed from intergenic regions of the genome (Wang et al., 2017; Sanchita et al., 2020). Accumulating evidence revealed that lincRNAs have potential roles involved in pathogen-defense responses and abiotic stress. For example, lincRNA *XLOC_026030* in rice is involved in the biological response to Pi starvation, and its expression level has a significant upward trend on the third day after Pi starvation (Xu et al., 2016). LincRNAs in the soybean participate in stress response, signal transduction, and developmental processes (Golicz et al., 2017). In potato, there is high association between 17 lincRNAs and 12 defense-related genes, which suggest that lincRNAs have potential functional roles in defense responses (Kwenda et al., 2016).

Wheat is a major food crop, a staple food worldwide, and one of the sources of plant protein for humans (Dong et al., 2020). Increasing wheat yield by reducing the influence of biotic/abiotic stresses is still widely studied. The impact of plant diseases on wheat-growing regions is difficult to estimate (Morgounov et al., 2012). Powdery mildew (*Blumeria graminis* f. sp. *tritici*) disease could infect all aboveground tissues of wheat, especially in humid environment (Conner et al., 2003). Breeding and utilization of Pm-resistant varieties is the most cost-effective and environmentally acceptable approach to control damage caused by powdery mildew (Hu et al., 2020). High-throughput sequencing technology provides great convenience in RNA sequencing (RNA-seq) for transcriptome analysis, and has been applied to reveal the expression patterns of genes that respond to plant disease defense mechanisms and discover novel genes (Zhu et al., 2015; Zhang H. et al., 2016; Zhang J. C. et al., 2016). So far, a total of 89 resistance genes/alleles have been identified to confer resistance to powdery mildew in wheat (Dong et al., 2020). However, recent studies indicated that many resistance genes have lost their resistance to powdery mildew (Tan et al., 2019). Therefore, it is necessary to find new sources to resist powdery mildew.

Although the mechanism mediating wheat responses to the pathogens causing powdery mildew has been widely investigated for years, long intergenic noncoding RNAs (lincRNAs), which have been proven to regulate important processes in the stress responses of plants, are still poorly known in wheat against powdery mildew infection. In this work, the multi-study datasets from public RNA-seq bio-projects currently available for wheat have been analyzed to identify the potential expression pattern of lincRNAs in response to wheat powdery mildew infection. First, lincRNAs in wheat were identified using RNA-seq data from a previous time-series experiment in which plants were grown under infected or non-infected conditions (Zhang et al., 2014). Second, differential expression analysis of the identified lincRNAs was performed to obtain the lincRNAs that respond to powdery mildew infection. Third, based on genomic location analysis methods, mRNA–lincRNA target pairs were predicted and functional annotation of target genes was performed. Finally, to determine whether they play important roles in resisting or promoting powdery mildew infection, the expression patterns of eight mRNA–lincRNA target pairs were analyzed using qRT-PCR. This study will provide not only new ideas for further understanding the pathogenic mechanism of wheat powdery mildew but also information for a more comprehensive understanding of the molecular mechanism involved in wheat resistance to powdery mildew.

## Materials and methods

### Downloading the raw data

The RNA-seq data used in this study were from the NCBI SRA database (accession number PRJNA243835). The samples were leaves at the two-leaf stage of wheat seedlings, which are infected with two different fungi (*Puccinia striiformis* f. sp. *tritici* and *Blumeria graminis* f. sp. *tritici*). Samples were collected at 0 h, 24 h, 48 h, and 72 h after fungi infection. In this study, only the experimental data of wheat infection with powdery mildew were used.

### RNA-Seq reads mapping and transcriptome assembly

The Fastq-dump_v2.8.0 tool was used to convert SRA files (paired-end sequencing data) into paired-end FASTQ format. FASTQC_v0.11.9 (FastQC Quality Control, version 0.11.9) software was used to assess the quality of all generated FASTQ files (Xiao et al., 2015). Trim_Galore_v0.6.7 tool was used for quality trimming and adapter removal with the default parameters. Subsequently, the clean reads were then mapped to the wheat (version 2.1, International Wheat Genome Sequencing Consortium) reference genome with the default parameters by the software HISAT2_v4.8.2 (Pertea et al., 2016). The file containing all mapped readings for each sample was saved in SAM format. SAM files contain the alignment position of sequencing data on the reference genome and other relevant information (Li et al., 2009). The parameter “sort -o” in software samtools_v1.9 was used to convert SAM format files into sorted BAM files (Pertea et al., 2016). BAM files are in binary format and are often used in subsequent analyses, such as mutation detection and gene expression analysis (McKenna et al., 2010). Using StringTie_v2.1.7 software, all BAM files were assembled into one complete GTF file with the default parameters (Pertea et al., 2015). In addition, StringTie software can estimate the expression levels of genes and transcripts in all samples (Pertea et al., 2016).

### Identification of lincRNAs in wheat

**Figure 1:**
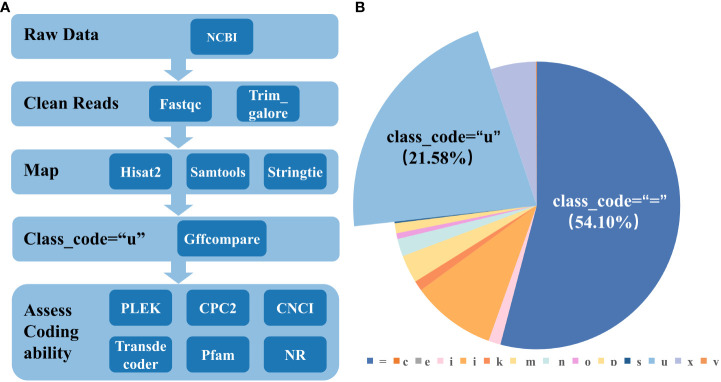
The identification process of lincRNA. **(A)** The specific identification process of lincRNA and the software and tools used at each step (Chen et al., 2019). **(B)** Location comparison of transcripts to reference genomes and the proportion of each species, as generated by GffCompare. Classification is based on previous studies (Pertea and Pertea, 2020).

Refer to previous research for lincRNA detection (Chen et al., 2019). Firstly, the parameter “- r” of gffcompare in StringTie was used to filter transcripts without the “u” character to ensure the preservation of intergenic transcripts (Pertea and Pertea, 2020). All of PLEK, CNCI, and CPC2 can be used to predict the encoding potential of transcripts. The transcript sequence with class_code = “ u “ was predicted by these three software to obtain three sets of transcripts that did not have coding capabilities. To reduce the impact of false positives, the intersection of the a, b, and c datasets was taken, and the final intersection was analyzed for subsequent analysis. To minimize the impact of false positives predicted by the software, the intersection of the three datasets was taken. The transcripts that were predicted to be non-coding in all three software were continued for subsequent analysis. LongOrfs is a core tool in software TransDecoder for predicting long open reading frames (ORFs) in transcripts. ORFs with a length of at least 100 amino acids are recognized by default. LincRNAs do not have long ORFs, so transcript sequences containing long ORFs were removed (Harrow et al., 2012). To further rule out the ability of the remaining sequences to encode proteins, the transcript sequence was translated into six possible protein sequences using the transeq command, each corresponding to a different reading frame. HMMER was used to identify whether these translated protein sequences contain specific protein domains, families, patterns, etc. (Finn et al., 2011). Protein sequences without special structures were used for subsequent analysis. Using BLAST search in the NR database, sequences similar to the query sequences can be found in known protein sequences. Protein sequences with an E-value greater than 1e-5 compared to known protein sequences were preserved. Retained transcripts as candidate lincRNAs (**Figure 1A**).

### Analysis of differentially expressed lincRNA (DE-lincRNA)

The GTF file from stringtie analysis contains information such as transcripts and their expressions. After converting them to the form required for DESeq2, differential expression analysis of the transcriptome data was performed using DESeq2 (Love et al., 2014). The online software Omicshare (https://www.omicshare.com/tools/) was used for differential expression analysis, and the parameter was *p* < 0.05.

### Prediction of adjacent target genes and functional annotation of target genes

Genomic location analysis method was used to predict adjacent target genes of lincRNA. In general, genes within a certain distance range are considered potential adjacent target genes. The distance of 100 kb is relatively close on the genomic scale. Genes closer to lincRNA may have a closer association and possibly functional relationship with lincRNA (Luo et al., 2016). Therefore, mRNA in the range of 100 kb expands in both upstream and downstream directions based on the location of differentially expressed lincRNA (DE-lincRNA) on chromosomes as possible target genes of lincRNA. GO and KEGG functional annotation of target genes was implemented using the online tool Omicshare (https://www.omicshare.com/tools/). The potential interaction between lincRNA and mRNA was plotted using software Cytoscape_v3.9.1. The software TBtools was used to generate the heat map of gene expression.

### Plant material and powdery mildew infestation

Wheat (Yangmai 20 variety) seeds were germinated in a greenhouse at 25 ± 2°C. After 2–3 days, healthy wheat seedlings were selected and transferred to a round flowerpot. There are three to five seedlings in each pot at the greenhouse. Seedlings about the two-leaf stage were inoculated with *Blumeria graminis* f. sp. *tritici* race E09 from infected wheat leaves under 18 ± 2°C (Guo et al., 2021). The infected wheat leaves were presented from Professor Lijun Yang (Institute of Plant Protection and Soil Science Hubei Academy of Agricultural Sciences). The wheat leaves were collected at 0 h, 24 h, 48 h, and 72 h after inoculation, and then maintained in a −80°C cryogenic refrigerator. This experiment used non-infected wheat leaves (0 h) as the control group.

### Total RNA extraction and qRT-PCR

Total RNA was extracted from wheat leaves (infected and non-infected leaves) with TRIzol reagent (Aidlab, Beijing, China) and was reverse transcribed using HiScript II Reverse transcriptase (Vazyme, Nanjing, China). Quantitative PCR was carried out in triplicate with the ChamQ SYBR Color qPCR Master Mix (Vazyme, Nanjing, China). The reactions were conducted in a 20-μL volume containing 10 μL of 2×ChamQ SYBR Color qPCR Master Mix, 0.4 μL of each primer (10 μmol/mL), 0.4 μL 50×ROX Reference Dye 1, and 2 μL of the cDNA, and the remaining double-distilled water was replenished to 20 μL. The annealing temperature of the primer was 60°C. Primers used in present study are listed in **Supplementary Table 3**.

## Results

### Characterization of lincRNA

**Figure 2:**
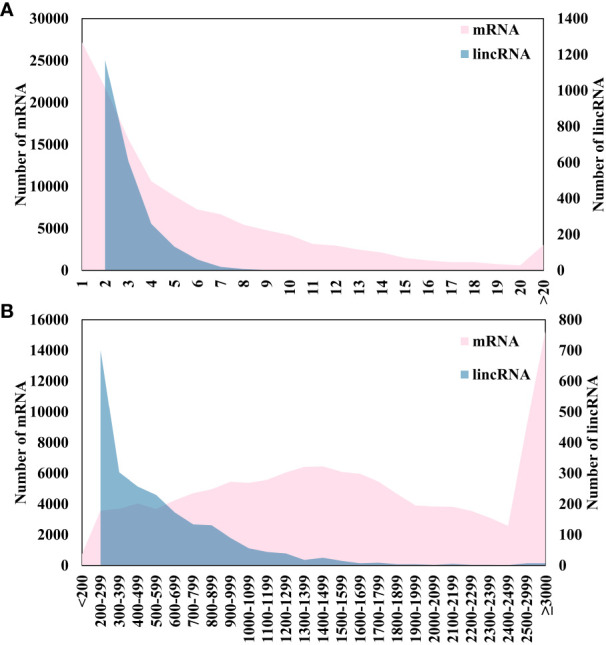
Analysis of the characteristics of lincRNA and mRNA. Pink represents mRNA and blue represents lincRNA. The primary ordinate was used for mRNA and the secondary ordinate was used for lincRNA. **(A)** Exon number analysis. **(B)** Gene length analysis.

After the low-quality sequencing fragments in the RNA seq data (**Supplementary Table 1**), wheat leaves under different stages of powdery mildew infection were filtered out, and 95.56% of the clean reads were successfully mapped. There are a total of 244,636 transcripts, of which 52,789 were recorded as class_code = “u”, accounting for 21.58% (**Figure 1B**), about half of the mRNA (class_code = “=“). After comparison with the genome and removal of coding sequences, a total of 2,266 lincRNAs were identified in wheat. The result of the number of exons of mRNA and lincRNA showed that the number of the number of exons gradually increases, and the number of mRNA and lincRNA showed a significant decrease (**Figure 2A**). In addition, there are also differences in sequence length between mRNA and lincRNA. The gene length of lincRNA ranged from 200 to 2,000 bp, and only a few lincRNAs had a length greater than 2,000 bp, and the number of lincRNA decreased with the increase of length. However, mRNA has a wide range of gene length distribution, and there are a large number of gene distributions in each length interval, and their number was not significantly different (**Figure 2B**).

### Identify the lincRNAs that can respond to powdery mildew infection

**Figure 3:**
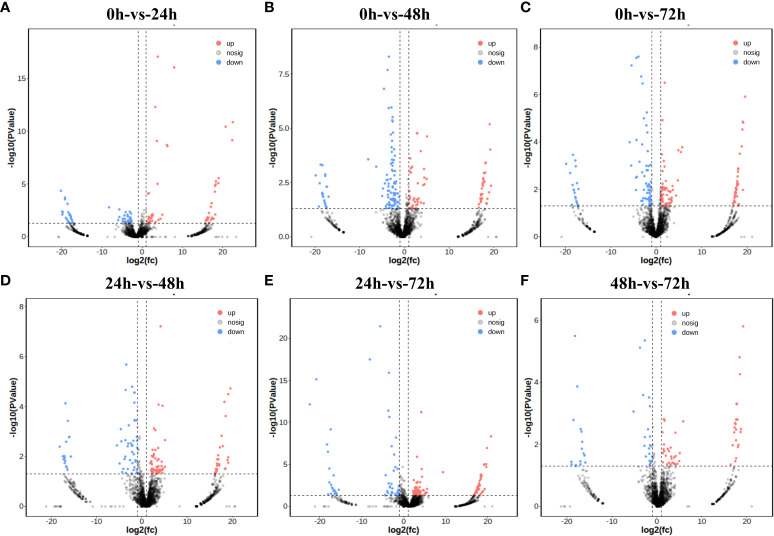
Differential expression analysis. Red indicates an upward expression and blue indicates a downward expression. Gray represents no significant difference. **(A–F)** Volcano plot for difference analysis for each comparison group.

According to the transcriptome data of four groups of wheat samples, the non-infected samples (0h) were compared with infected samples at three different time points (24h, 48h, and 72h). There were 107, 176, and 176 differentially expressed lincRNAs. Notably, although the number of DE-lincRNAs was the same in the latter two groups, the sequence of DE-lincRNAs was different (**Figures 3A–C**; **Supplementary Table 2**). Differential expression analysis was also performed in pairs among three infected samples of different time points, and 134, 154, and 85 DE-lincRNAs were obtained (**Figures 3D–F**; **Supplementary Table 2**). To identify the lincRNAs that can respond to powdery mildew infection, all the DE-lincRNAs in these six comparison groups were combined and repeated DE-lincRNAs in different groups were removed, resulting in a total of 486 DE-lincRNAs that could respond to powdery mildew infection in wheat.

### Prediction of adjacent target genes

Considering the physical distance, the 100-kb distance falls within the close range on the genome scale, and relatively close genes may have a closer association and possible functional relationship with lincRNAs. From the perspective of regulatory scope, many regulatory sequences and elements are located in the upstream and downstream regions of genes. Therefore, the choice of a distance range of 100 kb in our study allows a more comprehensive consideration of the potential regulatory effect of lincRNA on genes close to it. A total of 1,062 adjacent genes were obtained by locating 486 lincRNAs within 100 kb of both 5’ and 3’ directions. This was not a one-to-one relationship, because a lincRNA responds to multiple coding genes nearby.

### Functional annotations of target genes

**Figure 4:**
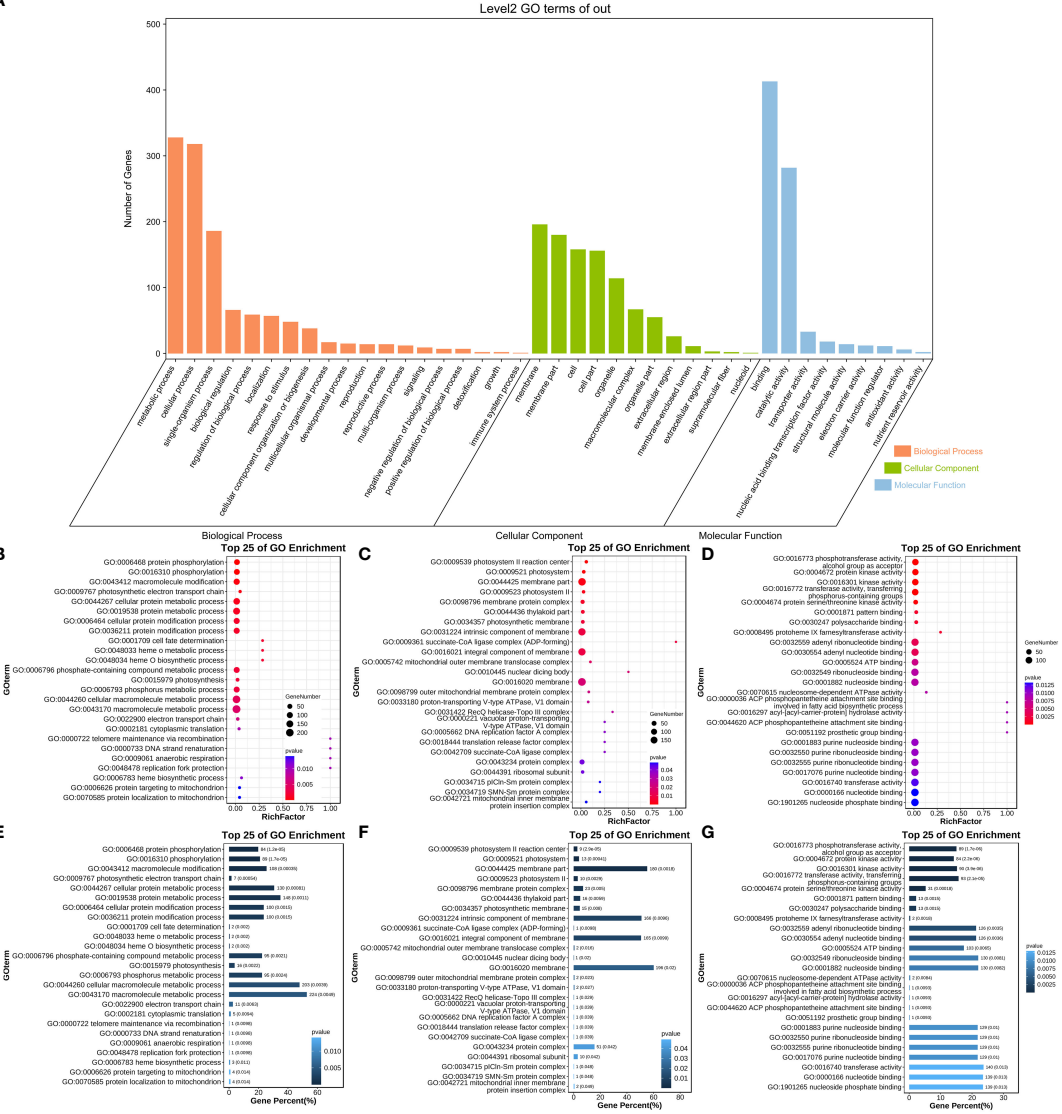
GO function analysis of neighboring target genes. **(A)** GO annotated secondary classification histogram of results. The abscissa represents the first-level classification of GO’s three ontology, the first of which is the biological pathway (BP), the second is the cellular component (CC), and the third is molecular function (MF). **(B–D)** GO-enriched bubble chart of the top 25 terms. The *x*-axis is the Rich Factor, the ratio of the number of genes enriched into the pathway by the set of selected genes to the number of genes enriched into the pathway by background genes. The *y*-axis is the name of the enriched pathway, arranged from smallest to largest according to *p*-value. The size of the dot indicates the number of genes, and the larger the dot, the more genes are enriched into that pathway. The color of the dot represents the level of the *p*-value, and the smaller the *p*-value, the more significant the pathway. **(E–G)** GO Enrichment Analysis Bar Chart. The abscissa is the proportion of the number of genes, and the ordinate is the GO Term and the details of each GO number. The different color depths in the figure represent different gene numbers, and the color gradually decreases from dark to light. From left to right, it corresponds to the specific classification of BP, CC, and MF.

**Figure 5:**
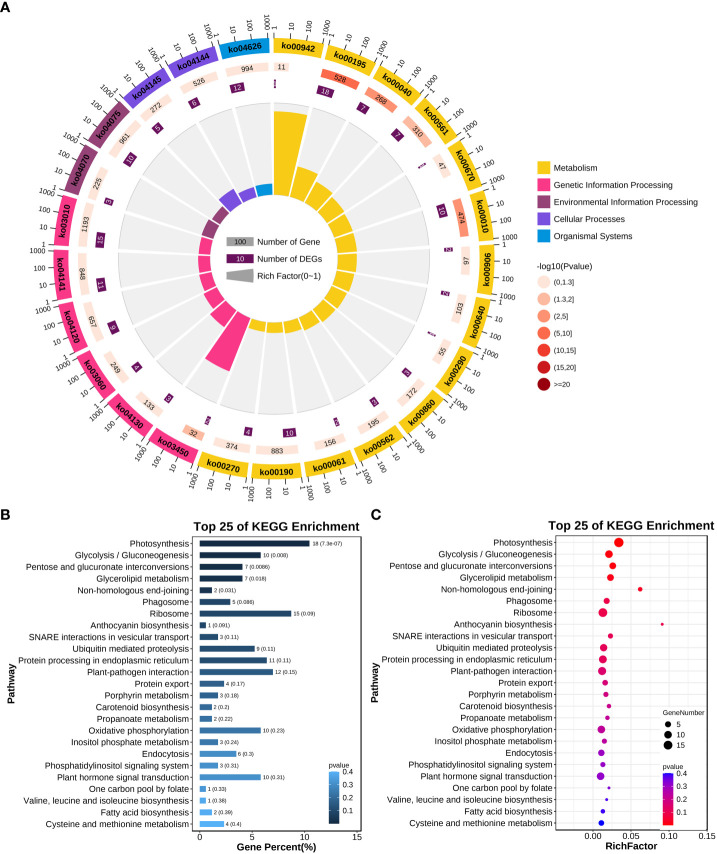
KEGG annotation results of adjacent target genes. **(A)** Enrichment circle chart. The first circle is the classification of enrichment. Outside the circle is the coordinate of the number of genes, and different colors represent different classifications. The second circle shows the total number of background genes in that category. The third circle is the number of selected genes enriched into the classification as well as the *p*-value. The more genes, the longer the bar, the smaller the value, the darker the color. **(B)** KEGG Enrichment Analysis Bar Chart. **(C)** KEGG-enriched bubble chart of the top 25 terms.

**Figure 6:**
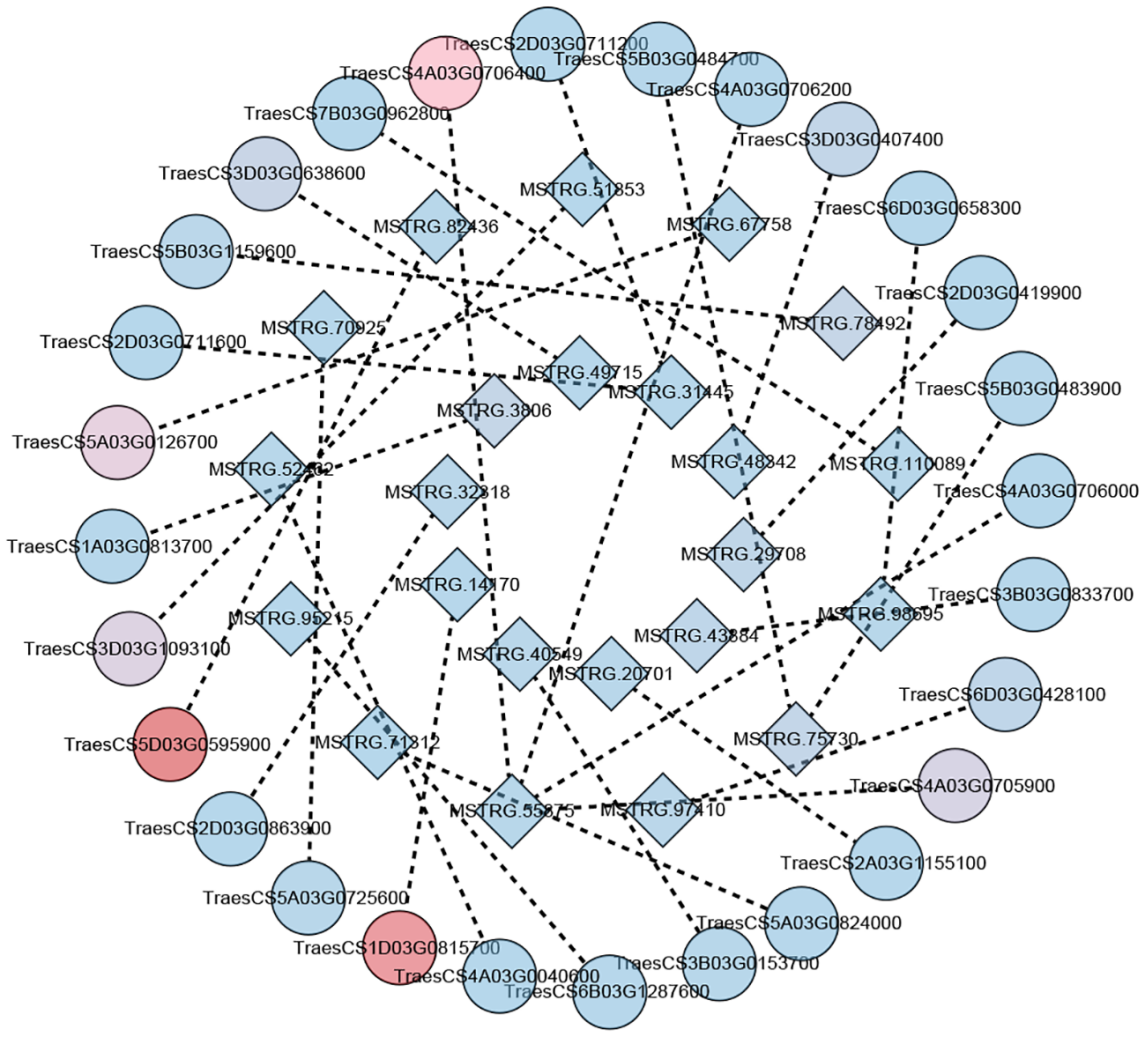
The correspondence between lincRNAs and mRNAs. The circle represents mRNAs and the square represents lincRNAs. Lines represent the presence of potential regulatory relationships between genes. The shade of color represents the level of gene expression. A change in color from blue to red indicates higher and higher levels of gene expression (the sum of expression at all time points was taken).

To clarify the function of the adjacent target genes and the biological processes involved, GO and KEGG functional annotations were used to analyze the function of target genes. A total of 727 target genes from the 1,062 adjacent target gene list were successfully mapped to terms in the GO database. There were three main categories for GO terms: Molecular Function, Cellular Component, Biological Process, and the number of genes was 592, 327, and 424, respectively (**Figure 4**; **Supplementary Table 4**). Some target genes were involved in more than one biological process. According to the KEGG annotation results (**Figure 5**), pathways related to the regulation of plant disease resistance mechanisms were selected, including fructose and mannose metabolism, MAPK signaling pathway-plant, plant hormone signal transduction, plant-pathogen, and starch and sucrose metabolism—five processes of interaction. A total of 28 genes involved in these processes were selected for further analysis (**Table 1**). The correspondence between lincRNAs and mRNAs is shown in **Figure 6**.

**Table 1:** Pathways associated with plant disease resistance and their corresponding genes.

Pathway	Pathway ID	Genes
Fructose and mannose metabolism (2)	ko00051	TraesCS2A03G1155100; TraesCS3D03G0638600
MAPK signaling pathway - plant (6)	ko04016	TraesCS4A03G0706000; TraesCS4A03G0705900; TraesCS5B03G0484700; TraesCS3D03G0407400; VTraesCS1A03G0813700; TraesCS5B03G0483900
Plant hormone signal transduction (10)	ko04075	TraesCS3B03G0153700; TraesCS2D03G0863900; TraesCS5A03G0126700; TraesCS5B03G0484700; TraesCS3D03G1093100; TraesCS4A03G0040600; TraesCS2A03G1155100; TraesCS1A03G0813700; TraesCS5B03G0483900; TraesCS3B03G0833700
Plant-pathogen interaction (12)	ko04626	TraesCS6B03G1287600; TraesCS5B03G1159600; TraesCS4A03G0706000; TraesCS4A03G0705900; TraesCS5A03G0725600; TraesCS7B03G0962800; TraesCS3D03G0407400; TraesCS5A03G0824000; TraesCS6D03G0428100; TraesCS5D03G0595900; TraesCS2D03G0419900; TraesCS5B03G0483900
Starch and sucrose metabolism (6)	ko00500	TraesCS2D03G0711200; TraesCS4A03G0706200; TraesCS2D03G0711600; TraesCS4A03G0706400; TraesCS3D03G0638600; TraesCS6D03G0658300

The numbers in parentheses represent how many genes of the pathway were included.

### Analysis of expression patterns of disease-resistant lincRNAs and their target genes

**Figure 7:**
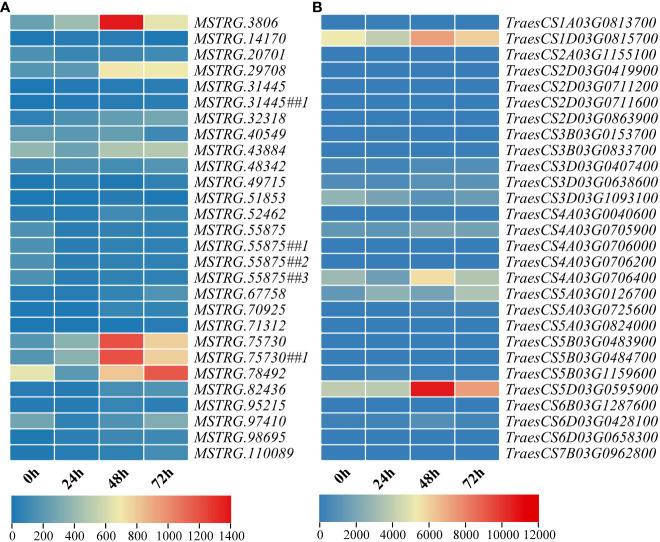
Heat map of expression levels of genes involved in the regulation of plant disease resistance mechanisms at four different time points (0 h, 24 h, 48 h, and 72 h). **(A)** Expression of 23 lincRNAs at different time points. **(B)** Expression of 28 mRNAs at different time points. To demonstrate the one-to-one correspondence of mRNAs to lincRNAs, several lincRNAs are repeated.

**Figure 8:**
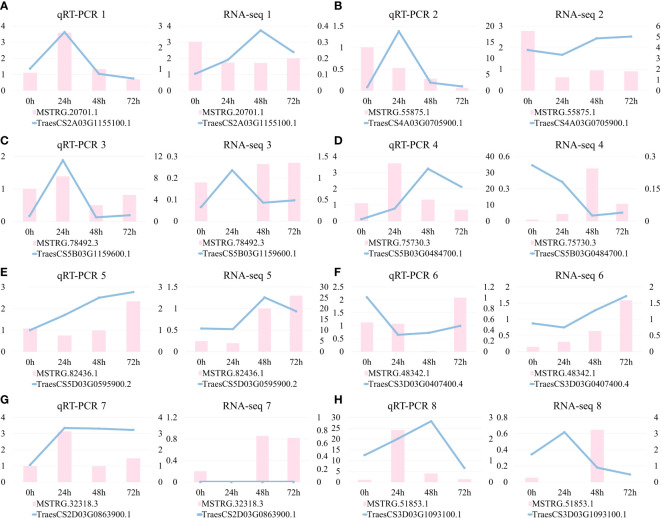
Analysis of expression patterns of lincRNAs and neighboring target genes. **(A–H)** The experimental and predicted results of eight pairs of mRNA–lincRNA correspond in turn. The figure on the left is plotted from the results of qRT-PCR and the figure on the right is plotted based on the gene expression obtained from RNA-seq data.

Since the target genes are identified by the genomic location analysis method, more than one mRNA is contained near a lincRNA. A total of 23 lincRNA–mRNA pairs were formed between 28 target genes and their nearby lincRNAs (**Supplementary Table 5**). There was clear evidence for higher expression of mRNAs than lincRNAs (**Figure 7**). Among them, eight pairs of genes with high expression were selected for expression pattern analysis. There were four pairs of mRNA whose changes in expression showed the same trend as the predicted results: the first, third, sixth, and eighth pairs (**Figures 8A, C, F, H**). The results of the expression pattern analysis of lincRNA show that the experiments of the second, fourth, fifth, and eighth pairs were consistent with the predicted results (**Figures 8B, D, E, H**). From the results of qRT-PCR, changes in the expression of mRNA in the first, third, fifth, and seventh pairs correlated with changes in lincRNA (**Figures 8A, C, E, G**). The expression patterns of the second, fourth, sixth, and eighth pairs were not significantly correlated (**Figures 8B, D, F, H**). As expected, some lincRNAs may have potential regulatory relationships with their corresponding target genes. Both lincRNAs and their target genes with the same expression pattern showed an increase in expression levels. This phenomenon indicates that these genes may be susceptible genes for powdery mildew.

## Discussion

To obtain lincRNA in wheat that can respond to powdery mildew infection, four groups of sequencing data from non-infected wheat samples and infected wheat samples at three different time points were analyzed. All the significantly DE-lincRNAs were aggregated to obtain the lincRNAs in wheat in response to powdery mildew infection, totaling 486 lincRNAs. A similar study identified 283 DE-lincRNAs that were tightly correlated with the fungi-responsive lincRNAs in wheat, of which 254 DE-lincRNAs responded to the powdery mildew stress (Zhang H. et al., 2016). The difference between this study and previous studies is that we selected a new reference genome of wheat. There are subtle differences in the identification of lincRNA, and the identification criteria of previous studies are more stringent. The transcripts containing ORF greater than 300 bp were removed in this study, while the transcripts from previous studies were required not to contain ORF more than 150 bp. This may be the reason for the difference in prediction results for lincRNA. Our study analyzed the co-expression patterns of lincRNAs with their adjacent protein-coding genes, and previous studies analyzed the co-expression patterns of selected miRNAs targeting lincRNAs and functional genes (Zhang H. et al., 2016). The diversity of prediction methods contributes to a comprehensive understanding of the disease defense mechanism of wheat powdery mildew.

Possible expression correlations between lincRNAs and their adjacent target genes were assessed to further clarify the specific mode of action of lincRNA in response to powdery mildew infection. The method based on genomic location analysis suggests that genes within a certain distance near lincRNAs may be potential adjacent target genes (Ørom et al., 2010; Engreitz et al., 2016). In our study, a range of 100 kb was chosen, which is a common choice rather than an absolute value. Different lincRNAs may have different regulatory ranges, and the regulatory distance range may be affected by other factors (Ørom and Shiekhattar, 2013; Kopp and Mendell, 2018). Consistent with the expected results, some lincRNAs showed significant correlation with the expression patterns of their potential target genes, while others did not show any association. It should be noted that the existing prediction methods only make preliminary predictions, and further experimental verification and functional studies are required to find the real target genes of lincRNA.

LincRNAs are becoming a key regulatory factor in various cellular processes, but it is still difficult to clarify the function of individual lincRNA (Ransohoff et al., 2018). The function of adjacent target genes can be used as a reference to identify the function of lincRNA (Guttman et al., 2009). To further understand the specific functions of lincRNA in response to powdery mildew infection, GO and KEGG function annotations were performed on potential target genes of DE-lincRNAs. The annotation results help us to better understand the functions of lincRNA and their roles in biological processes. However, there are still some inevitable defects. GO functional analysis is usually the annotation of the whole genome, which may lead to the omission or masking of the functional information of some genes. The annotation information in the GO database is based on known functions and biological processes, but functional annotation may be limited for some genes that are not fully understood (Gene Ontology Consortium, 2019). Our study was limited by the fact that 335 genes have not yet been successfully annotated. This part of the bias will narrow the scope of research that matches our target genes. The KEGG database is also mainly based on known signaling and metabolic pathways, which have not yet covered all biological processes and may not account for some newly discovered functions (Hucka et al., 2003; Kanehisa et al., 2017).

The functional annotation information indicates that *TraesCS2A03G1155100* is involved in pathway “Fructose and mannose metabolism” and “Plant hormone signal transduction”. Fructose and mannose are part of the process of sugar metabolism in plant. Sugar signaling contributes to plant immune responses to pathogens, and may act as a signal molecule to induce plant defense responses to invading pathogens (Bolouri Moghaddam and Van den Ende, 2012). Based on the functional similarities, it is speculated that lincRNA *MSTRG.20701* with the same expression pattern as the genes mentioned above may regulate fructose and mannose metabolism and thus participate in plant defense responses. Both *TraesCS2A03G1155100* and *TraesCS2D03G0863900* are involved in the process of plant hormone signal transduction. Numerous studies have shown that plants can produce hormones for disease resistance and defense (Yan et al., 2023). In addition, *TraesCS5D03G0595900* and *TraesCS5B03G1159600* participate in plant–pathogen interaction. This information provides a reference for further understanding the role of lincRNA in response to powdery mildew infection. However, the exact mechanisms of lincRNA in plant immunity require further research for confirmation.

## Data availability statement

The original contributions presented in the study are included in the article/**Supplementary Material**. Further inquiries can be directed to the corresponding authors.

## Author contributions

PC: Writing – original draft. YW: Data curation, Writing – original draft. ZM: Validation, Writing – review & editing. XX: Methodology, Supervision, Writing – review & editing. DM: Writing – review & editing. LY: Resources, Writing – review & editing.
